# Association filtering and generative adversarial networks for predicting lncRNA-associated disease

**DOI:** 10.1186/s12859-023-05368-z

**Published:** 2023-06-05

**Authors:** Hua Zhong, Jing Luo, Lin Tang, Shicheng Liao, Zhonghao Lu, Guoliang Lin, Robert W. Murphy, Lin Liu

**Affiliations:** 1 School of Information Science, Yunnan Normal University, Kunming, China; 2 State Key Laboratory for Conservation and Utilization of Bio-resource, School of Ecology and Environment and School of Life Sciences, Yunnan University, Kunming, China; 3 Key Laboratory of Educational lnformation for Nationalities Ministry of Education, Yunnan University, Kunming, China; 4 School of Medicine, Yunnan University, Kunming, China; 5 Reptilia Zoo and Education Centre, 2501 Rutherford Rd., Vaughan, ON L4K 2N6 Canada

**Keywords:** LncRNA-disease association prediction, Generative Adversarial networks, Filtering associations, Negative sampling

## Abstract

**Background:**

Long non-coding RNA (lncRNA) closely associates with numerous biological processes, and with many diseases. Therefore, lncRNA-disease association prediction helps obtain relevant biological information and understand pathogenesis, and thus better diagnose preventable diseases.

**Results:**

Herein, we offer the LDAF_GAN method for predicting lncRNA-associated disease based on association filtering and generative adversarial networks. Experimentation used two types of data: lncRNA-disease associated data without lncRNA sequence features, and fused lncRNA sequence features. LDAF_GAN uses a generator and discriminator, and differs from the original GAN by the addition of a filtering operation and negative sampling. Filtering allows the generator output to filter out unassociated diseases before being fed into the discriminator. Thus, the results generated by the model focuses only on lncRNAs associated with disease. Negative sampling takes a portion of disease terms with 0 from the association matrix as negative samples, which are assumed to be unassociated with lncRNA. A regular term is added to the loss function to avoid producing a vector with all values of 1, which can fool the discriminator. Thus, the model requires that generated positive samples are close to 1, and negative samples are close to 0. The model achieved a superior fitting effect; LDAF_GAN had superior performance in predicting fivefold cross-validations on the two datasets with AUC values of 0.9265 and 0.9278, respectively. In the case study, LDAF_GAN predicted disease association for six lncRNAs-H19, MALAT1, XIST, ZFAS1, UCA1, and ZEB1-AS1-and with the top ten predictions of 100%, 80%, 90%, 90%, 100%, and 90%, respectively, which were reported by previous studies.

**Conclusion:**

LDAF_GAN efficiently predicts the potential association of existing lncRNAs and the potential association of new lncRNAs with diseases. The results of fivefold cross-validation, tenfold cross-validation, and case studies suggest that the model has great predictive potential for lncRNA-disease association prediction.

## Introduction

Biologists first discovered the existence of long non-coding RNA [1] (lncRNA) in 1990s, which opened new doors for biomedical research. Long non-coding RNAs, which are more than 200 nucleotides in length, are involved in numerous biological processes, such as chromatin modification, cell proliferation, and transcriptional regulation [2]. A relationship exists between lncRNA and disease, such that mutations can lead to many diseases, such as lung cancer [3], cardiovascular diseases [4], and neurodegenerative diseases [5]. Therefore, the study of lncRNA-disease association can clarify the functions of lncRNA, and facilitate the prevention, diagnosis and treatment of human diseases. Unfortunately, biological experiments to prove the association between lncRNA and diseases are often costly, and this and time constitute major obstacles. Alternatively, computational methods can efficiently study the association of lncRNA with diseases. Current popular methods of research involve two categories: machine learning and predictive biological networks.

Recently, machine learning is a leading edge as exemplified by bioinformatics. Zhao et al. [6] developed a computational method based on a simple Bayesian classifier of lncRNA association data and a genome that achieved excellent results. However, their classifier requires on negative samples, which affects the model’s performance. Lan et al. [7] integrated multiple data sources of lncRNA and diseases, and used multiple approaches to calculate lncRNA and disease similarity for different data sources. They used a SVM classifier and organized it into a web server LDAP, by feeding RNA sequences into LDAP to make predictions. Biswas et al. [8] offered a method that used a non-negative decomposition matrix and multiple association data and expression profile data. Their low-rank computational model well described the association between two-dimensional matrices. In addition, an idea has been adopted by many studies that diseases or lncRNAs with similar properties may have the same association object. Chen et al [9]. constructed a semi-supervised framework, LRLSLDA, based on lncRNA and disease similarity. Advantageous, the semi-supervised model did not entirely rely on data labels. Although the method discovered potential associations without negative samples, the model has many parameters, and the choice of parameters will inevitably affect the prediction results. Finally, Chen et al. [10] developed two semantic similarity-based models, LNCSIM1 and LNCSIM2, and organically combined them with the LRLSLDA [9] model, which significantly improved model performance.

Biological networks usually build various association or similarity networks. The most common methods are based on lncRNA disease-association, lncRNA similarity, and disease similarity networks. For example, RWRlncD [11] uses lncRNA functional similarity networks and random wandering (RWR) to make a prediction, but its biggest drawback is that it cannot be applied to disease prediction without known associations. Geng et al. [12] constructed a heterogeneous network of lncRNAs, miRNAs, and diseases to discover potential associations. The greatest advantage of the heterogeneous network is that features of multiple nodes can be fused to make predictions more accurate. Yao et al. [13] constructed a multilayer composite network (LncPriCNet) on multiple interactions data. They used a RWR method to mine potential associations, and LncPriCNet still maintained advantages in the face of insufficient information on lncRNAs due to the support of the multilayer composite network. lncRDNetFlow [14] integrated multiple source networks to predict lncRNA disease associations, and in the absence of known associations.

Both types of methods require lncRNA-disease association data. Because verified biological experimental association data are limited, researchers must use lncRNA expression profiles, tissue specificity, gene location, etc. to predict associations. Li et al. [15] used gene locations to predict the association of lncRNAs with vascular disease. The drawback of their method is that it is limited by gene location information, and because there is no guarantee that the lncRNAs have adjacent genes, even if there are adjacent genes, they are not necessarily associated. Liu et al. [16] proposed using disease and lncRNA expression profiles that can be independent of known lncRNA-disease relationships, but the few associated gene records limit its application.

Recent attention has focused Generative Adversarial Networks (GANs). GANs consist of a generator and a discriminator, which can be trained to generate samples extremely similar to the original data [17]. GANs have no dimensional requirement on the input of the generator, and only require a gradient back-propagation to train the model, avoiding the use of complex Markov chains. This iterative approach has spread to the natural language processing and recommendation system. GANs has the advantage of semi-supervised learning. Most of the data in the recommendation system yield only positive feedback, which is ideal for training with semi-supervised learning. Currently, many GAN recommendation models have emerged, such as IRGAN [18], GraphGAN [19], PSGAN [20], APL [21], and CFGAN [22]. Numerous studies have shown that these models obtain superior results in the recommendation domain compared to baseline models. In this paper, lncRNA-disease association data and user-item recommendation data have similar properties, i.e., prediction of unknown associations based on known association data. So it is reasonable to assume that for the same sparse lncRNA-disease association data, GAN-based association prediction algorithms have significant advantages over supervised learning. Notably, Du et al. [23] offered LDA-GAN for lncRNA-disease association prediction. It uses the Gumbel-SOFTMAX technique to construct separable processes to simulate discrete sampling.

Inspired by LDA-GAN and CFGAN. The application of LDA-GAN in lncRNA disease association prediction guided our study, but the inability to directly back-propagate gradients during model training also posed many inconveniences. In contrast, CFGAN with real-valued vector adversarial training achieves better prediction results, which provides a new idea for our research. Unlike CFGAN, our model needs to process lncRNA sequence data and obtain effective implicit feature information, so network structures that can extract sequence features need to be introduced in the design of generators and discriminators. Meanwhile, considering the sequence characteristics of lncRNA nucleotide sequences, this paper combined Doc2Vec and fully connected neural network to optimize the generator and discriminator, so that lncRNA sequence features is able to more accurately participate in the generation and determination of lncRNA-disease association. we offer LDAF_GAN, a method for lncRNA-associated disease prediction based on association filtering and GANs. The filtering involves a dot product of the generated results of the generator with real data (retaining the part of the association matrix that corresponds to 1 in real data). Thus LDAF_GAN only focuses on associated data. Negative sampling assumes that values of 0 in the association matrix have no association, and the remaining values have unknown associations. By adding a regular term to the loss function, the generator cannot generate all-1 vectors, which ultimately improves the generative power of LDAF_GAN. Experimental results reveal that LDAF_GAN has fivefold cross-validation AUC values over 91% and tenfold cross-validation AUC values over 92% on four trial datasets. Further, disease associations for six common lncRNAs predicted by LDAF_GAN reveal that accuracy can achieve 100%, 80%, 90%, 90%, 100%, and 90% for H19, MALAT1, XIST, ZFAS1, UCA1, and ZEB1-AS1, respectively.

## Methods

### Generative adversarial networks

GAN architecture [17] of the LDAF_GAN model, which was built to fit our data, was not based on a fixed structured model, but rather an adversarial framework. The structure of the sub-model depended on the type of data. Parameter definitions are listed in Table 1.

**Table 1:** Parameter definitions for the LDAF_GAN model

$\theta$	Parameters of the generator
$\phi$	Parameters of the discriminator
*z*	Random noise
$ld\_r_{g}$	LDAF_GAN generates data
$c\_seq$	lncRNA sequence characteristics
$\{\}$	Connection symbols
$ld\_seq\_r_{g}$	LDAF_gan_seq generates data
$ld\_real$	Real data
$fua\_real$	Data for filtering operations
$ld\_r^{'}_{g}$	LDAF_gan filtered results
$ld\_seq\_r^{'}_{g}$	LDAF_gan_seq filtered results
*G*	Generators
*D*	Discriminator
$fua\_real\_sample$	Filtered data after negative sampling
$\alpha$	Adjustable parameters for regular terms
$N\_sample$	Number of negative sampling samples

#### Generative network

LDAF_GAN mainly consisted of a generative and discriminative networks. The former was a multilayer, fully connected neural network denoted as $G(\theta )$. When LDAF_GAN was trained on dataset1, the input was represented as $G(\theta ,z)$, where $\theta$ was the parameter of the generator and z the random noise. Next, the output of *G* was expressed as $ld\_r_{g}$ (i.e., represents the result of the random noise *z* input to the generator and then output by the softmax function), which had the same dimension as the noise dimension. When LDAF_GAN was trained on dataset2, the input was represented as $G(\theta ,\{z, c\_seq\})$, where $c\_seq$ denoted the lncRNA sequence feature, $``\{\}''$ denoted the connection noise *z*, and the lncRNA sequence feature was $c\_seq$, and the output of *G* was $ld\_seq\_r_{g}$.

#### Discriminative network

Discriminator network was also a multilayer fully connected neural network that had the same input dimensions, but the output dimension was 1. Accordingly, $D(\phi )$ was used to represent the discriminator, where $\phi$ was the parameter of the discriminator. In LDAF_GAN, every iteration of the discriminator required the input of real and generated data. The generated data were represented as either $ld\_r_{g} \bigodot fua\_real$ or $ld\_seq\_r_{g} \bigodot fua\_real$ (where operation $``\bigodot ''$ was used for filtering and $fua\_real$ was consistent with the real data). Then, generated data were fed into the discriminator as either $D(\phi , G(\theta ,z))$ or $D(\phi , G(\theta , \{z, c\_seq\}))$. Meanwhile, $ld\_real$ ($ld\_real$ represents the Boolean matrix composed of real association data) or $\{ld\_real,c\_seq\}$ was input to *D*. Finally, the model give feedback to *G* by calculating the gap between the generated data and the real data, and the gradient back propagation was adjusted to the *G* network to generate new data that were closer to the original data distribution.

#### LncRNA sequence feature extraction

**Fig. 1:**
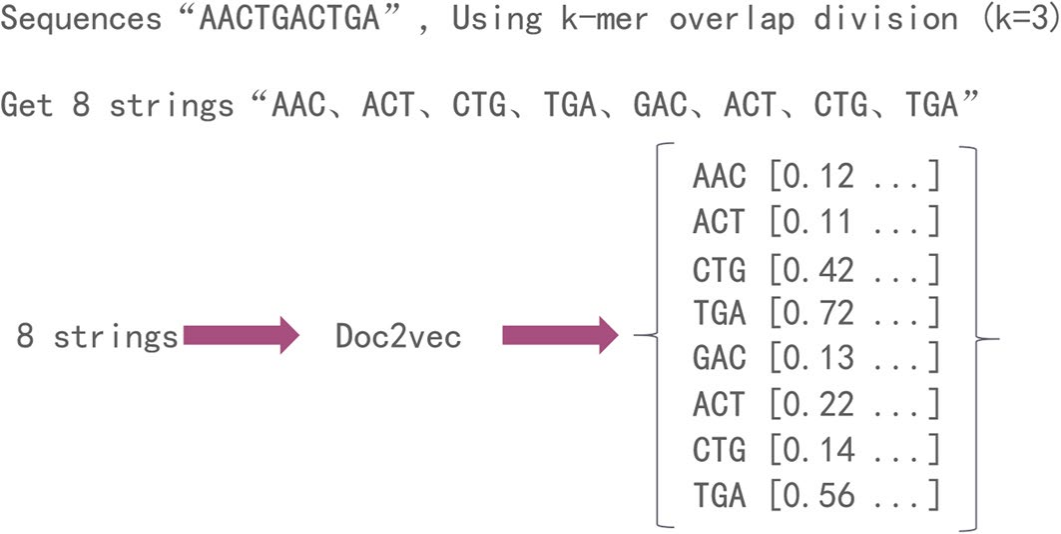
lncRNA feature extraction

The lncRNA sequence is composed of four nucleotides, “A”, “G”, “C” and “T”. The traditional k-mer method obtains the feature of sequences by counting the frequency of nucleotide occurrences. However, the k-mer ignores the order of nucleotides, thus we would like to take the nucleotide order into consideration in this study. Firstly, we adopt the idea of k-mer to divide the sequence into several sub-strings of length k; secondly, we adopt the Doc2vec model to obtain the vector expression of each string; finally, these strings are combined vertically into a matrix in order to obtain the features of the sequence. An example is shown in Fig. 1.

### LDAF_gan

**Fig. 2:**
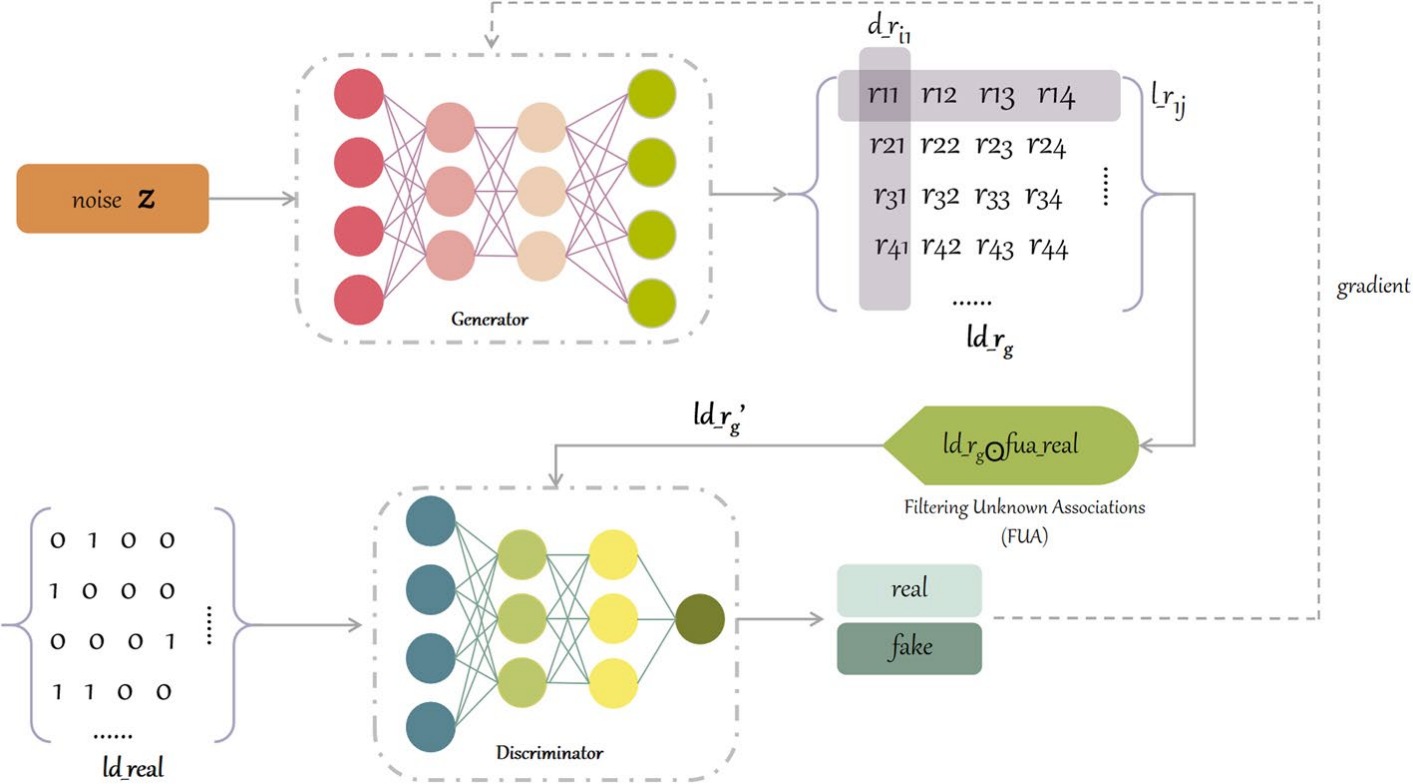
Architecture of LDAF_gan

The sub-model LDAF_gan of LDAF_GAN was trained using associated data without lncRNA sequence features. Upon combining the generative and discriminative networks, we completed a generative adversarial network. We followed the common GAN [17] to construct the LDAF_GAN model. The main structure involved generator G and the discriminator *D*, but for the output of *G* we employed a simple filtering operation; the final model architecture is shown in Fig. 2.

Following the input of random noise, generator $G(\theta ,z)$ yielded a matrix of lncRNA-disease association where $\theta$ was a hyperparameter of *G* and *z* denoted noise. The row vector of the matrix represents the likelihood that a particular lncRNA is associated with all diseases, and the column vector represents the likelihood that a disease is associated with all lncRNAs. Each value of matrix was interpreted as the association likelihood of a lncRNA with a predicted disease. Each value of vector was interpreted as the association of an lncRNA with a predicted disease. The softmax function normalized the association between (0,1) to get $ld\_r_{g}$. Next, filtering (Filtering Unknown Associations, FUA) eliminated the effect of unknown associations on the generated results. The filtering operation is to dot product the output of the generator with the negative sampling matrix of real lncRNA-disease association, and the filtering will retain the values corresponding to the positive and negative samples in the generated results, which makes the discriminator feedback more accurate. After filtering, ld_rg’ is obtained, which was denoted as $ld\_r_{g} \bigodot fua\_real$. Then, $ld\_r_{g}'$ was fed into the discriminator together with real data $ld\_real$ as $D(\phi , G(\theta ,z))$ and $D(\phi ,ld\_real)$, and the output value indicated the probability of accuracy. Finally, *D* was fed back to *G*, which made adjustments, where *G* minimized the probability of a false result; the loss function of *G* was expressed as Eq.  (1).

1 $$\begin{aligned} J^{G}=\min _{\theta } E_{z\sim P_{noise\left( z \right) } }\left[ log\left( 1-D\left( G\left( z \right) \right) \right) \right] \end{aligned}$$

where *z* denotes the noise vector that obeys the distribution of noisy data $P_{noise}$. Correspondingly, *D* improved discriminatory capabilities by maximizing the gap between the real data and the generated data. Thus, the loss function of *D* was expressed as Eq. (2).

2 $$\begin{aligned} J^{D}=\max _{\phi } E_{x\sim P_{real\left( x \right) } }\left[ logD\left( x \right) \right] + E_{z\sim P_{noise\left( z \right) } } \left[ log\left( 1- D\left( G\left( z \right) \right) \right) \right] \end{aligned}$$

where *x* denotes the vector of real data, following the real data $P_{real}$ distribution. *G* and *D* were represent in Eq. (3).

3 $$\begin{aligned} \min _{G} \max _{D}V\left( G,D \right) =E_{x\sim P_{real\left( x \right) } } \left[ logD\left( x \right) \right] + E_{z\sim P_{noise\left( z \right) } }\left[ log\left( 1- D\left( G\left( z \right) \right) \right) \right] \end{aligned}$$

### LDAF_gan_seq

**Fig. 3:**
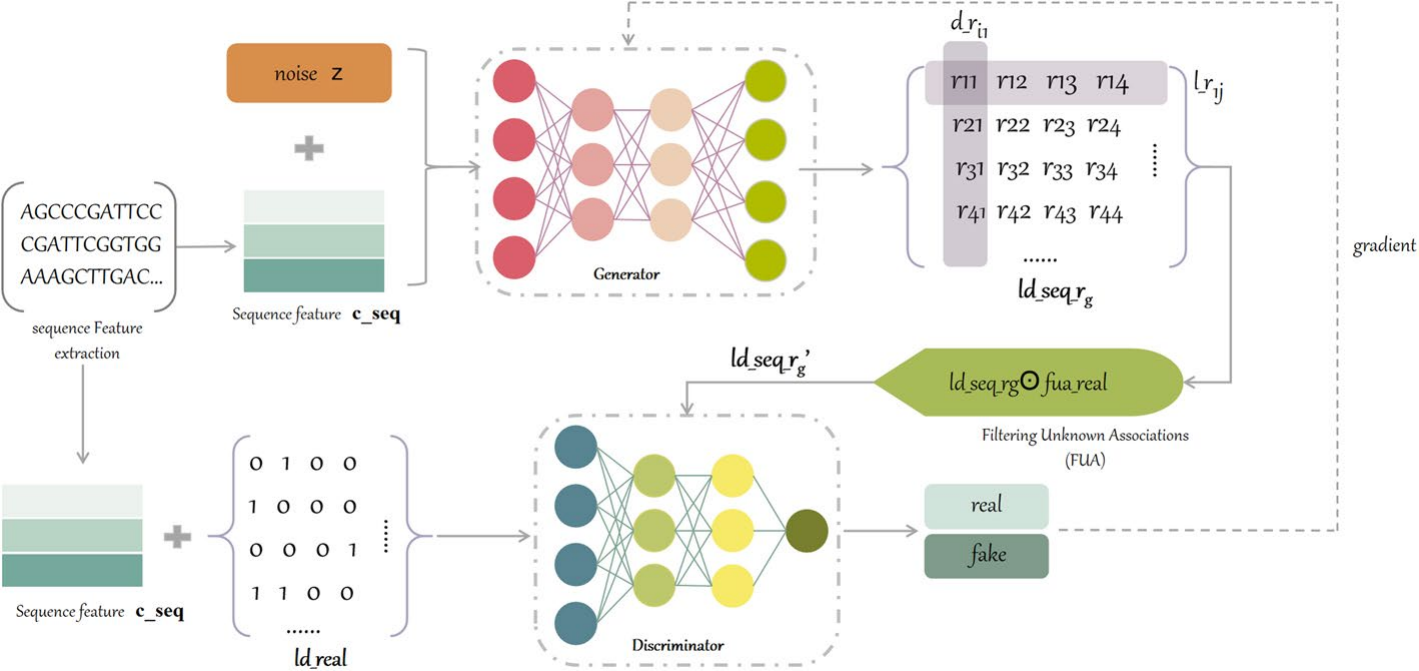
Sub-model LDAF_gan_seq

Sub-model LDAF_gan_seq was trained with fused lncRNA sequence data. LDAF_gan_seq differed slightly from LDAF_gan via slightly adjusting *G* and *D* network dimensions of LDAF_gan. The overall model is shown in Fig. 3.

Features of lncRNA sequences, mainly composed of “A”, “G”, “C” and “T”, were extracted using the methods mentioned in above. It is worth noting that for lncRNA sequence features, our model specifically constructs a network module in the generator to extract the implicit features of lncRNA sequences, and the same network layer is set in the discriminator, which constitutes the overall model structure of sequence feature processing, lncRNA-disease association generation and discrimination. Random noise was then connected with sequence features as the input of *G*, which is represented by $G(\theta ,\{z,c\_seq\})$ (Fig. 2), where $``\{\}''$ denotes connecting the noise *z* with the feature $c\_seq$, and outputting the same dimension as the noise after passing *G*. Subsequently, the softmax function was used to normalize $ld\_seq\_r_{g}$, followed by the FUA operation $ld\_seq\_r_{g} \bigodot fua\_real$, filtering out the unknown association data to get $ld\_seq\_r_{g}'$. Real data $ld\_real$ and generated data $ld\_seq\_r_{g}'$ were fed into *D*, and the results are expressed as $D(\phi ,ld\_real)$ and $D(\phi ,G(\theta ,\{z,c\_seq\}))$, where $\phi$ is the hyperparameter of *D*. The output was judged to be the probability of accuracy. The loss function of *G* in LDAF_GAN was expressed as Eq. (4).

4 $$\begin{aligned} J^{G}=\min _{\theta } E_{z\sim P_{noise\left( z \right) } }\left[ log\left( 1-D\left( G\left( z,c\_seq \right) \right) \right) \right] \end{aligned}$$

Then, the loss function of D was shown in Eq. (5).

5 $$\begin{aligned} J^{D}=\max _{\phi } E_{x\sim P_{real\left( x \right) } }\left[ logD\left( x,c\_seq \right) \right] + E_{z\sim P_{noise\left( z \right) } } \left[ log\left( 1- D\left( G\left( z,c\_seq \right) \right) \right) \right] \end{aligned}$$

### Negative sampling

Sections 1.2 and 1.3 introduce the GAN-based LDAF_GAN approach to prediction. The greatest drawback in our approach is the absence of negative samples for the association data. Values of 0 in the matrix do not differentiate between no association and an association waiting to be predicted. In LDAF_GAN, the generation of all-1 vectors, which is the same result as the original data after filtering, is meaningless, therein necessitating a negative sampling strategy. Partial negative sampling in the unknown association data (matrix value of 0) gives value to unknown associations, which are denoted as no association. To avoid generating all vectors with values of 1, a regular term is added to the loss function in the training of *G*. The loss function of *G* is changed slightly (taking the fused lncRNA sequence feature model as an example), and the loss of *G* after negative sampling is represented as follows:

6 $$\begin{aligned} \begin{aligned} J^{G}&=E_{z\sim P_{noise\left( z \right) } }\left[ log\left( 1-D\left( G\left( z,c\_seq \right) \right) \right) + \alpha \sum _{i}^{} \left( x_{li } - \hat{x }_{li} \right) ^{2} \right] \\&={\textstyle \sum _{lnc}^{}} \left[ log\left( 1-D\left( \left( ld\_seq\_r_{g} \cdot fua\_real \right) \mid c\_seq \right) \right) + \alpha \sum _{i}^{} \left( x_{li } - \hat{x }_{li} \right) ^{2} \right] \end{aligned} \end{aligned}$$

where $x_{li}$ denotes the negative sampling result on the original data and $\hat{x}_{li}$ is negative sampling result on $fua\_real$. Considering the associated and unassociated terms in the training process of *D*, the negative sampling results must be as close to 0 as possible, while the true association is as close to 1 as possible to give feedback information to *G*. Accordingly, the loss function of *D* is adjusted as follows:

7 $$\begin{aligned} \begin{aligned} J^{D}&=\max _{\phi } E_{x\sim P_{real\left( x \right) } }\left[ logD\left( x\mid c\_seq \right) \right] \\&\quad+E_{z\sim P_{noise\left( z \right) } } \left[ log\left( 1- D\left( \left( ld\_seq\_r_{g}\cdot fua\_real\_sample \right) \mid c\_seq \right) \right) \right] \\&= - {\textstyle \sum _{lnc}^{}}\left[ logD\left( x\mid c\_seq \right) \right] \\&\quad +\left[ log\left( 1-D\left( \left( ld\_seq\_r_{g} \cdot fua\_real\_sample \right) \mid c\_seq \right) \right) \right] \end{aligned} \end{aligned}$$

### Model evaluation

To facilitate a comparison between LDAF_GAN and other models, we used fivefold and tenfold cross-validations. The former divides the samples into five uniformly disjoint parts, one as the test set and the remaining four as the training set. We then conducted five experiments in turn. The latter cross-validation divided the samples into ten uniformly disjoint parts, one part as the test set and the remaining nine as the training set, and we conducted ten experiments. The AUC was the area enclosed by the curves and axes in the coordinate system, with *FPR* as the horizontal coordinate and *TPR* as the vertical coordinate. This was used to evaluate the predictive performance of the model. The *AUPR* value was the area enclosed by the curve and the axis in the coordinate system, composed of recall as the horizontal coordinate and precision as the vertical coordinate. This evaluated the overall performance of the model, and was calculated as follows:

$$\begin{aligned} FPR= & {} \frac{FP}{TN+FP},TPR=\frac{TP}{TP+FN}\\ recall= & {} \frac{TP}{TN+FN},precision=\frac{TP}{TP+FP} \end{aligned}$$

where *TP* denoted the probability of positive samples being correctly predicted as positive samples, and *FN* denoted the probability of positive samples being incorrectly predicted as negative samples. *FP* denoted the probability of negative samples being incorrectly predicted as positive samples, and *TN* denoted the probability of negative sample being correctly predicted as negative samples.

## Results

### Comparison with other state-of-the-art methods

#### Experimental setting and datasets

This experiment was implemented on the PyTorch platform. To demonstrate the performance of the model, LDAF_GAN was applied to four datasets. First, fivefold cross-validation and tenfold cross-validation were performed on two publicly available datasets taken from BiGAN [24]: LncRNADisease 2.0 [25] and Lnc2Cancer v2.0 [26]. Further, lncRNADisease 2.0 [25] contained 19,166 lncRNAs and 529 diseases, then 205,959 lncRNA-disease association data were finally obtained; there were 9254 lncRNA-disease associations in the Lnc2Cancer dataset, which contained 2659 lncRNAs and 216 diseases. Second, cross-validation was performed on our constructed datasets dataset 1 and dataset 2, both of which contained 5213 associations with 1301 lncRNAs and 497 diseases, while there were 1301 corresponding lncRNA sequences in dataset 2 and none in dateset 1 (lncRNA sequence data from the database Refseq [25])).

#### LncRNADisease dataset comparison experiment

**Fig. 4:**
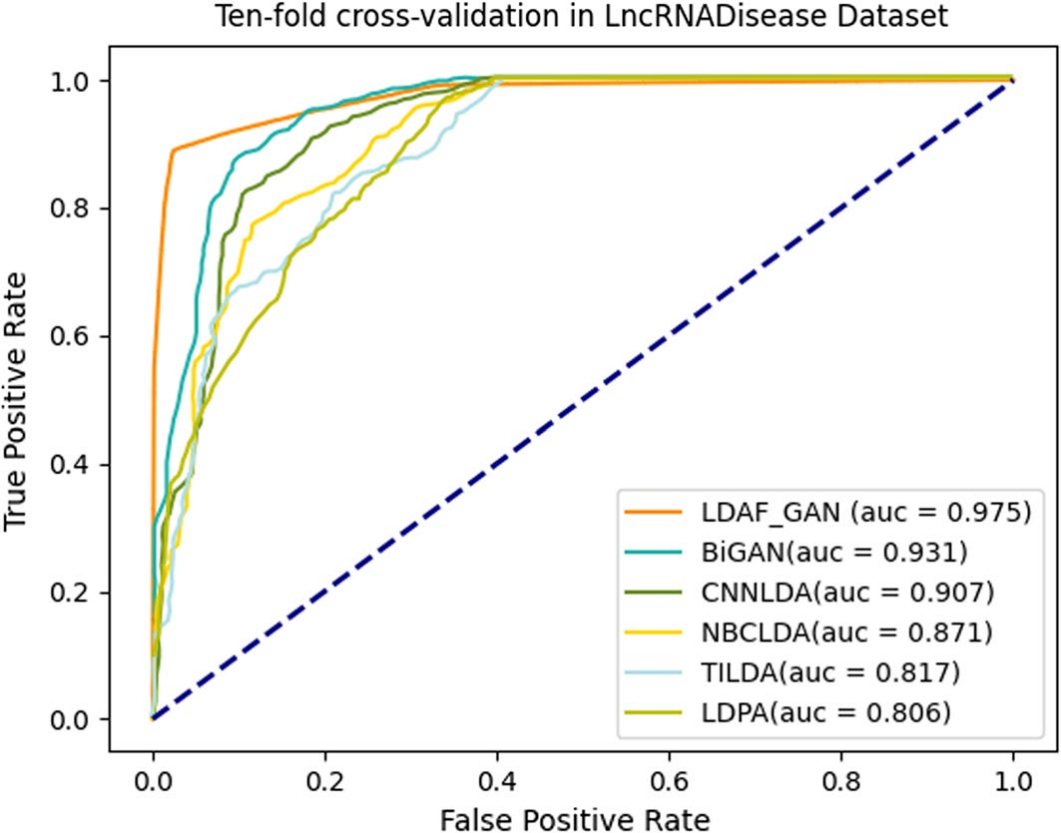
Ten-fold cross-validation on LncRNADisease dataset (results except for LDAF_GAN taken from Yang et al. [24])

Firstly, we compared several methods, such as BiGAN [24], CNNLDA [26], NBCLDA [29], TILDA [27] and LDAP [7] on the LncRNADisease 2.0 [25] dataset, and the tenfold cross-validation ROC curves were shown in Fig. 4. Experimental results showed that the LDAF_GAN method on the LncRNADisease dataset was significantly more effective than several other methods, and the AUC value reached about 0.975. The results demonstrated that the semi-supervised learning of GAN is very effective for lncRNA-disease association prediction even when only positive samples were available.

#### Lnc2Cancer dataset comparison experiments

**Fig. 5:**
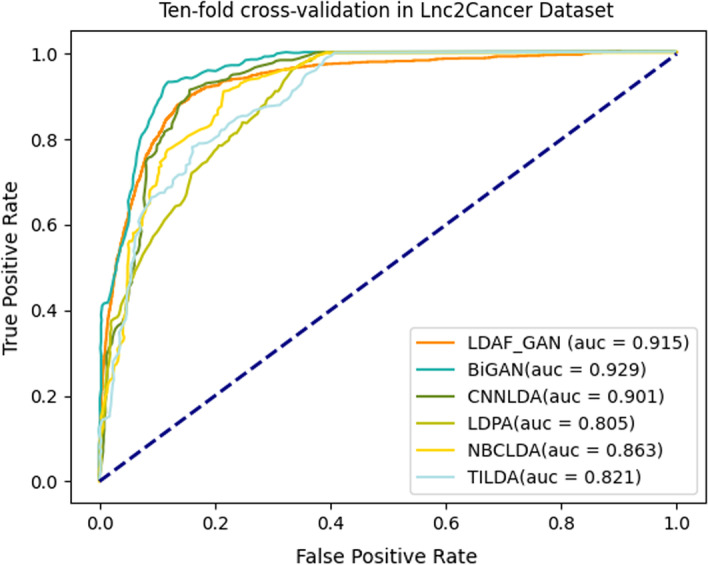
Ten-fold cross-validation on Lnc2Cancer dataset (results except for LDAF_GAN taken from Yang et al. [24])

LDAF_GAN was compared with BiGAN [24], CNNLDA [26], NBCLDA [29], TILDA [27] and LDAP [7] on the Lnc2Cancer v2.0 [26] dataset. The AUC value reached 0.915, and ROC curves under tenfold cross validation were shown in Fig. 5. LDAF _GAN achieved the best results among these models, and was second only to BiGAN [25]. However, our model did not utilize lncRNAs or a diseases similarity network, and relied on association data alone. For the prediction of a new lncRNA node, LDAF_GAN only required the lncRNA sequence features without calculating the similarity vector again.

#### Experiments on all data sets

**Fig. 6:**
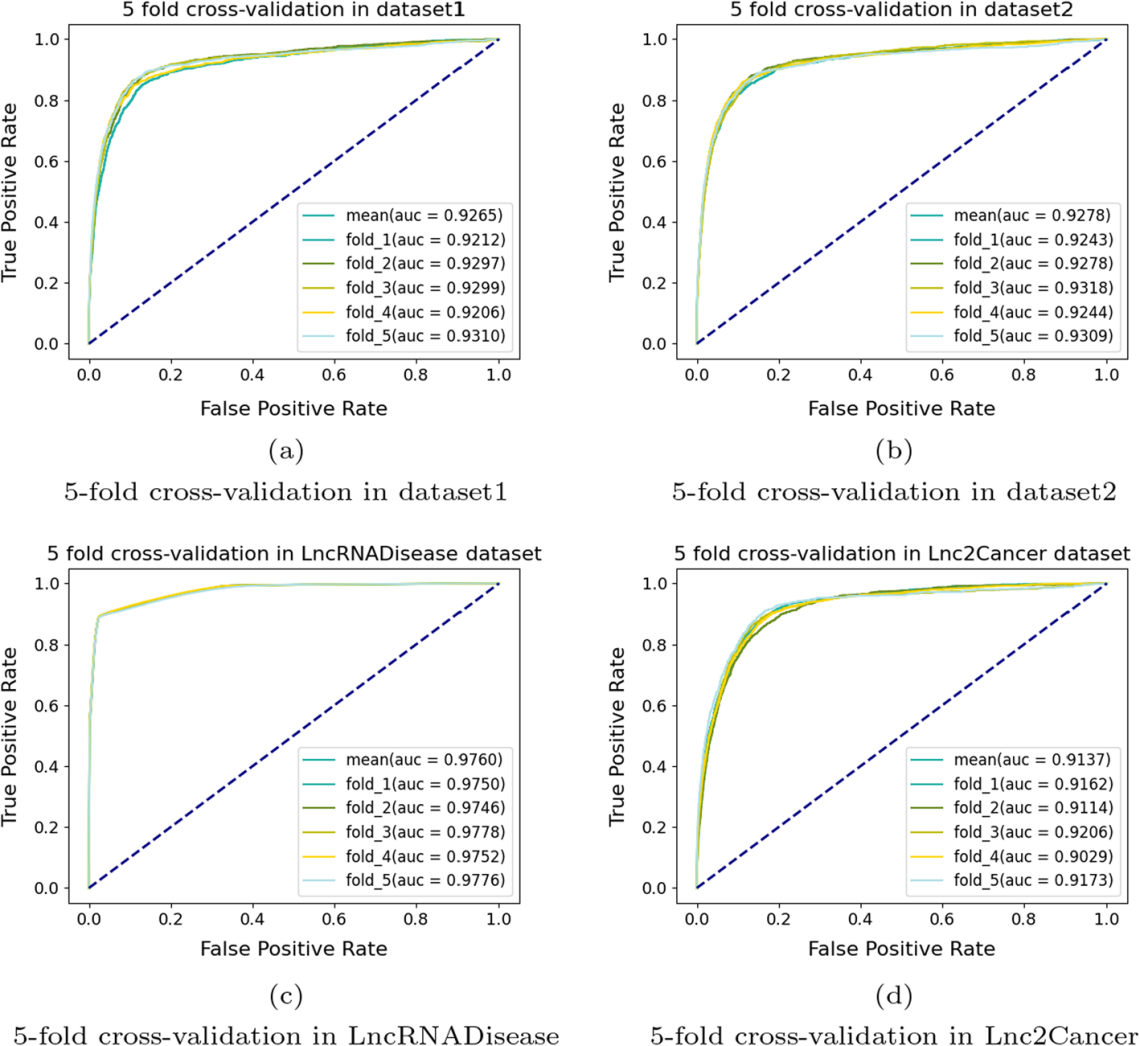
Five-fold cross-validation results of LDAF_GAN on four datasets

Finally, LDAF_GAN was cross-validated on dataset 1 and dataset 2, and then divided into two sub-models: LDAF_gan and LDAF_gan_seq. The LDAF_gan model was constructed on dataset 1, while the LDAF_gan_seq model was constructed on dataset 2. The results of LDAF_GAN on both datasets showed that LDAF_GAN predicted well not only the association data without sequence features, but also the multimodal data with fused sequence features. Our fivefold cross-validation results were shown in Fig. 6, where Fig. 6a showed the results of LDAF_GAN on dataset 1; the average AUC value obtained was 0.926. Figure 6b showed the fivefold cross-validation results of LDAF_gan_seq on dataset 2, and the average AUC value was 0.928. Figure 6c showed the fivefold cross-validation results of LDAF_GAN on the LncRNADisease [25] dataset, and Fig. 6d the fivefold cross-validation results of LDAF_gan on the Lnc2Cancer [26] dataset. Finally, all results of the LDAF_GAN validation on three datasets without lncRNA sequence features were shown in Table 2, including the mean AUC values and mean AUPR values under five- and tenfold cross-validation. LncRNADisease [25] dataset reached an average AUPR value of 0.45, Lnc2Cancer [26] dataset an average AUPR value of 0.15, and our dataset an average AUPR value of 0.15. LDAF_GAN achieved excellent predictions on several datasets, which was initially designed for training on continuous values, and while still achieving excellent predictions on discrete values. The negative sampling strategy used by LDAF_GAN helped the model to selectively generate results close to 0 or 1, thus avoiding misclassification caused by generating all-1 vectors.

**Table 2:** Experimental results of LDAF_GAN on different datasets (Note: dataset1 is a prediction without serial correlation data)

	AUC/AUPR	LncRNADisease	Lnc2Cancer	dataset1
10-fold cross-validation	AUC	0.9752	0.9145	0.9192
	AUPR	0.4502	0.1553	0.1457
5-fold cross-validation	AUC	0.9760	0.9137	0.9265
	AUPR	0.4861	0.1493	0.1876

### Case studies

We used lncRNAs H19, MALAT1, XIST, ZFAS1, UCA1, and ZEB1-AS1 to verify LDAF_GAN. The two sub-models based on LDAF_GAN have two prediction methods, both of which is able to predict the disease associated with a specific lncRNA. Based on LDAF_gan, the known association vector (i.e., the vector containing only 0 and 1) of lncRNA(e.g., H19) is fed into the generator, and after passing through the softmax function, an association vector will be output, whose elements are between (0,1). Disregarding the known associated diseases among them, the top-ranked corresponding diseases in the predicted associations are selected as the final prediction results. Based on LDAF_gan_seq, a random noise is connected with the sequence features of a certain lncRNA (e.g., ZFAS1) and fed into the trained generator. Finally, an association vector also is output through softmax function, and the top-ranked diseases are selected as the final prediction result. The predicted disease association on H19, MALAT1, and XIST reached 100$\%$, 80$\%$, and 90$\%$, respectively (based on experimental validations from the Lnc2Cancer [18] database) (Tables 3, 4, 5, respectively). We singled out lncRNA ZFAS1, UCA1, and ZEB1-AS1, which were not included in the training data, and used the lncRNA sequence features of ZFAS1, UCA1, and ZEB1-AS1 as the input of LDAF_GAN to generate the top 10 ranked diseases. The prediction accuracy value reached 90$\%$, 100$\%$, and 90$\%$, respectively (Table 6, 7, 8, respectively).

**Table 3:** The top 10 predicted H19 associated with diseases

LncRNA	Disease	Rank	Evidence
H19	Hepatocellular carcinoma	1	Lnc2Cancer
	Gastric cancer	2	Lnc2Cancer
	Breast cancer	3	Lnc2Cancer
	Colorectal cancer	4	Lnc2Cancer
	Esophageal squamous cell carcinoma	5	Lnc2Cancer
	Glioma	6	Lnc2Cancer
	Nasopharyngeal cancer	7	Lnc2Cancer
	Prostate cancer	8	Lnc2Cancer
	Ovarian cancer	9	Lnc2Cancer
	Lung cancer	10	Lnc2Cancer

**Table 4:** The top 10 predicted MALAT1 associated with diseases

LncRNA	Disease	Rank	Evidence
MALAT1	Hepatocellular carcinoma	1	Lnc2Cancer
	Gastric cancer	2	Lnc2Cancer
	Breast cancer	3	Unconfirmed
	Colorectal cancer	4	Lnc2Cancer
	Non small cell lung cancer	5	Lnc2Cancer
	Esophageal squamous cell carcinoma	6	Lnc2Cancer
	Glioma	7	Lnc2Cancer
	Lung adenocarcinoma	8	Lnc2Cancer
	Prostate cancer	9	Lnc2Cancer
	Cervical cancer	10	Unconfirmed

**Table 5:** The top 10 predicted XIST associated with diseases

LncRNA	Disease	Rank	Evidence
XIST	Hepatocellular carcinoma	1	Lnc2Cancer
	Breast cancer	2	Lnc2Cancer
	Colorectal cancer	3	Lnc2Cancer
	Non small cell lung cancer	4	Lnc2Cancer
	Esophageal squamous cell carcinoma	5	Lnc2Cancer
	Nasopharyngeal cancer	6	Lnc2Cancer
	Glioma	7	Lnc2Cancer
	Lung adenocarcinoma	8	Lnc2Cancer
	Osteosarcoma	9	Lnc2Cancer
	Cervical cancer	10	Unconfirmed

**Table 6:** The top 10 predicted ZFAS1 associated with diseases

LncRNA	Disease	Rank	Evidence
ZFAS1	Colorectal cancer	1	dataset 1
	Hepatocellular carcinoma	2	dataset 1
	Breast cancer	3	dataset 1
	Non small cell lung cancer	4	dataset 1
	Prostate cancer	5	dataset 1
	Gastric cancer	6	dataset 1
	Astrocytoma	7	Unconfirmed
	Esophageal squamous cell carcinoma	8	dataset 1
	Glioma	9	dataset 1
	Ovarian cancer	10	dataset 1

**Table 7:** The top 10 predicted UCA1 associated with diseases

LncRNA	Disease	Rank	Evidence
UCA1	Breast cancer	1	dataset 1
	Hepatocellular carcinoma	2	dataset 1
	Non-small cell lung cancer	3	dataset 1
	Colorectal cancer	4	dataset 1
	Gastric cancer	5	dataset 1
	Cervical cancer	6	dataset 1
	Prostate cancer	7	dataset 1
	Astrocytoma	8	dataset 1
	Ovarian cancer	9	dataset 1
	Glioma	10	dataset 1

**Table 8:** The top 10 predicted ZEB1-AS1 associated with diseases

LncRNA	Disease	Rank	Evidence
ZEB1-AS1	Breast cancer	1	dataset 1
	Hepatocellular carcinoma	2	dataset 1
	Colorectal cancer	3	dataset 1
	Gastric cancer	4	dataset 1
	Non-small cell lung cancer	5	dataset 1
	Astrocytoma	6	dataset 1
	Prostate cancer	7	dataset 1
	Cervical cancer	8	Unconfirmed
	Glioma	9	dataset 1
	Stomach cancer	10	dataset 1

## Discussion

Our lncRNA-associated disease prediction model LDAF_GAN,which is based on association filtering and Generative Adversarial Networks, fuses lncRNA sequence features to achieve prediction. Compared with several other prediction models, LDAF_GAN is stable and achieves superior performance. As a semi-supervised learning GAN, LDAF_GAN achieves good results even when only positive samples are available. In particular, the prediction of new lncRNAs does not require associated data, and the data distribution captured by GAN during training can support the prediction of new nodes. Thus, the method can achieve superior results both on the prediction of the original nodes and the prediction of new nodes. Further, excellent prediction results derives from two key points: filtering and negative sampling. Filtering allows the model to focus only on the parts with known associations, while negative sampling avoids pattern collapse due to the generation of all-1 vectors, which are also crucial for training. Finally, case studies show that LDAF_GAN accurately predicts disease associations for lncRNAs with known associations and lncRNA nodes without known associations but with lncRNA sequences. Nevertheless, some future developments may enhance predictions. For example, we adopt a negative sampling strategy, yet there is no guarantee that the negative samples are unassociated, which affects judgment of the model. For diseases, the model performance will be improved once appropriate disease features are incorporated.

## Conclusion

LncRNAs play an important role in biological life processes, and discovering potential associations between lncRNAs and diseases facilitates better diagnoses and prevention of diseases. To this end, we offer a method for lncRNA-associated disease prediction based on association filtering and Generating Adversarial Networks, called LDAF_GAN. Experiments use two types of datasets: association data, and association data with fused lncRNA sequence features. Sub-model LDAF_gan is useful for cases of only association data. Three association data validate this approach, and the results show that the fivefold cross-validation AUC values of LDAF_gan are 0.9760, 0.9137 and 0.9265. For data with sequence features, sub-model LDAF_gan_seq is applicable. It has an average fivefold cross-validation AUC value of 0.9278. Validation results from several datasets show that LDAF_GAN can achieve excellent results and high accuracy predictions. LDAF_gan_seq can make predictions in the absence of lncRNA-disease association, which is valuable in the absence of known associations; accurate predictions can be achieved using sequence features of lncRNAs as input. The validation results of LDAF_GAN on two publicly available and our own datasets verify credibility of the model. The correct prediction of most of the top 10 diseases by LDAF_GAN for six lncRNAs demonstrates its effectiveness.

## Data Availability

The LDAF_GAN source code is available at https://github.com/ZhonghuaYNNU/LDAF_GAN LncRNADisease v2.0: http://www.rnanut.net/lncrnadisease/index.php/home/info/download. Lnc2Cancer: http://www.bio-bigdata.net/lnc2cancer/download.html. dataset1: http://www.cuilab.cn/lncrnadisease and http://www.bio-bigdata.net/lnc2cancer/download.html. dataset2: https://lncipedia.org/ and https://www.ncbi.nlm.nih.gov/refseq/.

## Abbreviations

$ld\_r_{g}$: LDAF_GAN generates data
$c\_seq$: lncRNA sequence characteristics
$ld\_seq\_r_{g}$: LDAF_gan_seq generates data
$$ld\_real$$: Real data
$fua\_real$: Data for filtering operations
$ld\_r^{'}_{g}$: LDAF_gan filtered results
$ld\_seq\_r^{'}_{g}$: LDAF_gan_seq filtered results
$fua\_real\_sample$: Filtered data after negative sampling
$N\_sample$: Number of negative sampling samples

## References

[1] Qian X, Zhao J, Yeung PY, Zhang QC, Kwok CK (2019). Revealing lncRNA structures and interactions by sequencing-based approaches. Trends Biochem Sci.

[2] Mercer TR, Dinger ME, Mattick JS (2009). Long non-coding RNAs: insights into functions. Nat Rev Genet.

[3] De Kok JB, Verhaegh GW, Roelofs RW, Hessels D, Kiemeney LA, Aalders TW, Swinkels DW, Schalken JA (2002). Dd3(pca3), a very sensitive and specific marker to detect prostate tumors. Cancer Res.

[4] Klattenhoff C, Scheuermann J, Surface L, Bradley R, Fields P, Steinhauser M, Ding H, Butty V, Torrey L, Haas S (2013). Braveheart, a long noncoding RNA required for cardiovascular lineage commitment. Cell.

[5] Faghihi MA, Modarresi F, Khalil AM, Wood DE, Sahagan BG, Morgan TE, Finch CE, Georges SLI, Kenny PJ, Wahlestedt C (2008). Expression of a noncoding RNA is elevated in Alzheimer’s disease and drives rapid feed-forward regulation of beta-secretase. Nat Med.

[6] Zhao T, Xu J, Liu L, Bai J, Xu C, Xiao Y, Li X, Zhang L (2015). Identification of cancer-related lncRNAs through integrating genome, regulome and transcriptome features. Mol BioSyst.

[7] Lan W, Li M, Zhao K, Liu J, Wu F-X, Pan Y, Wang J (2016). LDAP: a web server for lncRNA-disease association prediction. Bioinformatics.

[8] Ashis Kumer Biswas MK (2015). Inferring disease associations of the long non-coding RNAs through non-negative matrix factorization. Netw Model Anal Health Inform Bioinform.

[9] Chen X, Yan GY (2013). Novel human lncRNA-disease association inference based on lncRNA expression profiles. Bioinformatics.

[10] Yan C, Luo C, Ji W, Zhang Y, Dai Q (2015). Constructing lncRNA functional similarity network based on lncRNA-disease associations and disease semantic similarity. Sci Rep.

[11] Sun J, Shi H, Wang Z, Zhang C, Zhou M (2014). Inferring novel lncRNA-disease associations based on a random walk model of a lncRNA functional similarity network. Mol BioSyst.

[12] Xia G, KaiJian H (2021). Mirna-disease association prediction based on network representation learning method. Appl Res Comput.

[13] Yao Q, Wu L, Li J, Yang L, Sun A, Li Z, He S, Feng F, Li H, Li Y (2017). Global prioritizing disease candidate lncRNA via a multi-level composite network. Sci Rep.

[14] Zhang J, Zhang Z, Chen Z, Deng L (2019). Integrating multiple heterogeneous networks for novel lncRNA-disease association inference. IEEE/ACM Trans Comput Biol Bioinf.

[15] Jianwei LI, Gao C, Wang YC, Wei MA, Jian TU, Wang JP, Chen ZZ, Kong W, Cui QH (2014). A bioinformatics method for predicting long noncoding RNAS associated with vascular disease. Sci China.

[16] Liu MX, Chen X, Chen G, Cui QH, Yan GY (2014). A computational framework to infer human disease-associated long noncoding RNAS. PLoS ONE.

[17] Goodfellow I, Pouget-Abadie J, Mirza M, et al. Generative adversarial nets. Adv Neural Inf Process Syst 2014:2672–2680.

[18] Wang J, Yu L, Zhang W, Gong Y, Xu Y, Wang B, Zhang P, Zhang D. Irgan: a minimax game for unifying generative and discriminative information retrieval models. In: Proceedings of the 40th international ACM SIGIR conference on research and development in information retrieval (2017).

[19] Wang H, Jia W, Wang J, Miao Z, Guo M. Graphgan: graph representation learning with generative adversarial nets. In: The 27th ACM international conference (2017).

[20] Lu S, Dou Z, Xu J, Nie JY, Wen JR. Psgan: a minimax game for personalized search with limited and noisy click data. In: The 42nd international ACM SIGIR conference (2019).

[21] Sun Z, Wu B, Wu Y, Ye Y (2019). APL: adversarial pairwise learning for recommender systems. Expert Syst Appl.

[22] Chae DK, Kang JS, Kim SW, Lee JT. Cfgan: a generic collaborative filtering framework based on generative adversarial networks. In: The 27th ACM international conference (2018)

[23] Du B, Tang L, Liu L, Zhou W (2022). Predicting LncRNA-disease association based on generative adversarial network. Curr Gene Ther.

[24] Yang Q, Li X (2021). BiGAN: LncRNA-disease association prediction based on bidirectional generative adversarial network. BMC Bioinform.

[25] O’Leary NA, Wright MW, Brister JR, Ciufo S, Pruitt KD (2015). Reference sequence (RefSeq) database at NCBI: current status, taxonomic expansion, and functional annotation. Nucl Acids Res.

[26] Xuan P, Cao Y, Zhang T, Kong R, Zhang Z (2013). Dual convolutional neural networks with attention mechanisms based method for predicting disease-related lncRNA genes. Front Genet.

[27] Ping P, Wang L, Kuang L, Ye S, Iqbal MFB, Pei T. A novel method for LncRNA-disease association prediction based on an lncRNA-disease association network. IEEE/ACM Trans Comput Biol Bioinform. 2019;16(2):688–93. 10.1109/TCBB.2018.2827373 29993639

[28] Xuan P, Cao Y, Zhang T, Kong R, Zhang Z (2013). Dual convolutional neural networks with attention mechanisms based method for predicting disease-related lncRNA genes. Front Genet.

[29] Yu J, Ping P, Wang L, Kuang L, Li X, Wu Z (2018). A novel probability model for lncRNA-disease association prediction based on the Naïve Bayesian classifier. Genes.

[30] Ping P, Wang L, Kuang L, Ye S, Iqbal MFB, Pei T (2019). A novel method for LncRNA-disease association prediction based on an lncRNA-disease association network. IEEE/ACM Trans Comput Biol Bioinform.

